# Supplementary material for: Acupotomy in Korean Medicine Doctors: A Preliminary Survey on Experiences, Perceptions, and Clinical Usage Status
Source: Healthcare (Basel). 2023 Sep 18;11(18):2577. doi: 10.3390/healthcare11182577 (PMC10530791; doi:10.3390/healthcare11182577)
Supplement: Supplementary file 1 [file healthcare-11-02577-s001.zip › healthcare-2516983-supplementary.pdf]

Supplementary Video S1. Visual demonstration of the procedural steps of acupotomy. https://www.youtube.com/embed/vAUW25Wwu5I?feature=oembed

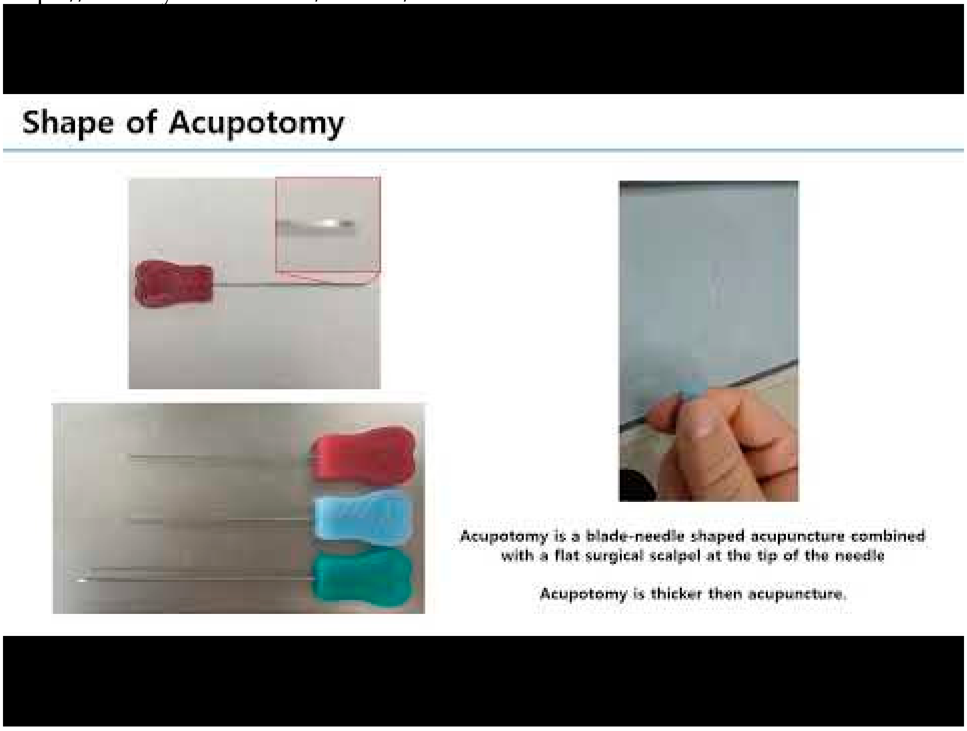

Supplement S1. Survey questionnaire.

Clinical usage status of acupotomy treatment

1. Please describe your career years of using acupotomy. [ ]
Ex: If it is 5 years, enter "5."

2. Select a commonly used blade width (diameter, in mm) in order of 1-3.
(When using only 1-2, you can select only 1-2. When selecting another option, please describe the specifications in the other columns.)

	1	2	3
0.4			
0.5			
0.6			
0.75			
0.8			
1			
1.2			
Others			
Don't know			

Others (be specific): [ ]

3. Select the body parts most frequently used for acupotomy treatment in order of 1-3.
(Please describe the specifications in the field ***Others*** below the table, when selecting Others.)

	1	2	3
Head and neck			
Face			
Back			
Lumbar and pelvis			
Chest			
Abdomen			
Upper extremities			
Lower extremities			
Others			

Others (be specific): [ ]

4. Please describe the disease most frequently treated in each area in the order of 1-3 selected in the above question.

[1] [ ]
[2] [ ]
[3] [ ]

5. Select the average depth of insertion.

① epidermis
② dermis
③ muscle
④ periosteum
⑤ don't know

6. Please describe the average number of insertion points per session. [ ]

7. Please describe the average number of up and down incision actions per point. [ ]

8. **Please describe the recommended inter-session interval days for patients.**[1] [ ]
   Ex) Enter only "1" for daily and "7" for weekly recommendations.

9. **When looking back on the past month, please select the total number of acupotomy treatment sessions per week on average.**
   Ex.) For 1 week, acupotomy treatment was considered nine times for three patients each.[2]
   (If you select 51 or more sessions, please describe the number of treatment sessions.)
① Less than five times
② 6-10
③ 11-20
④ 21-30
⑤ 31-40
⑥ 41-50
⑦ 51 or more: [ ]

10. **Please select the disinfection items you are using during the acupotomy procedure.**
   (Duplicate selection was allowed)
① Disposable acupotomy
② Sterilization wraps
③ Surgical masks
④ Surgical caps
⑤ Surgical gloves
⑥ Surgical gowns
⑦ Povidone-iodine
⑧ Alcohol
⑨ Boric acid
⑩ Antibiotics
⑪ Others (be specific): [ ]

11. **Please select the treatments used for pain relief.**
   (Duplicate selection was allowed)
① Surface anesthesia (anesthetic cream)
② Local anesthesia with conventional anesthetics (lidocaine, etc.)
③ Local anesthesia with herbal anesthetics (Sumsu pharmacopuncture, etc.)
④ Spinal anesthesia
⑤ Epidural anesthesia
⑥ Non-steroidal anti-inflammatory drugs
⑦ Oral herbal analgesics
⑧ Ice packs
⑨ Others (be specific): [ ]
⑩ None

12. **Please select the disinfection and treatment methods after the acupotomy.**
   (Duplicate selection was allowed)
① Disinfection and sterilization
② Dressing with sterile gauze
③ Band-aid application
④ Compression for hemostasis
⑤ Others (be specific): [ ]

The section is expanded upon incorporating feedback to enhance clarity and understanding.

---

[1] The section is expanded upon incorporating feedback to improve clarity and understanding.

[2] The revisions incorporated are in response to the feedback received.

13. Do medical imaging devices (such as sonography, C-arm, etc.) exist in the workplace to visualize the anatomical structures of patients?

① Yes
② No

14. Are you using a medical imaging device for safety and anatomy during the acupotomy procedure?

① Yes
② No

15. What is the most used medical imaging device during acupotomy?

① X-ray
② C-arm
③ Sonography
④ CT
⑤ MRI
⑥ Others (be specific): [ ]

Perception of the clinical applicability of the "pre- and post-acupotomy procedure safety checklist" guidelines

Although clinical treatment guidelines for acupotomy have not been developed yet, this paper presents a checklist for pre- and post-acupotomy treatment as a preliminary study. This section serves as a survey to identify areas that require improvement among the following items when developing future clinical guidelines.[3]

* The following is a checklist before and after the acupuncture procedure, which is suggested for safety.
[Checklist before the procedure]
Patient identity
Identification of contraindications
Vital signs: blood pressure, pulse, respiratory rate, body temperature
Position: supine position, prone position, lateral position, sitting position, etc.
Indication of exact point site
Anatomically safe treatment site

[Checklist during the procedure]
Vital signs observation: blood pressure, pulse, respiratory rate, body temperature
Patient response observation: nerve stimulation symptoms, pain during the procedure
Assessment of the appropriateness of the procedure in terms of needling depth and blade direction

[Checklist after the procedure]
Nerve damage: motor, sensory deficit
Blood vessel damage: bleeding, hematoma
Pain: constant pain
Vital signs: blood pressure, pulse, respiratory rate, body temperature
Patient education: pain control, preventing infection

Jo HG, Song MY, Yoon SH, et al. Proposal of Checklists for Patient Safety in Miniscalpel Acupuncture Treatment of Cervical and Lumbar Spine: Pilot Trial. *J Korean Med Rehabil.* 2018;28(1):61-72. doi:10.18325/jkmr.2018.28.1.61

16. Do you think that this item is necessary for safety before acupotomy?
(If there are more than two, please select multiple values.)
① Check the patient's identity and information (medication history, medical history, etc.).
② Identification of contraindications
③ Check vital signs (blood pressure, pulse, respiratory rate, body temperature)
④ Check the correct position according to the treatment site
⑤ Indication of exact point site
⑥ Confirmation of anatomical safe treatment site
⑦ None

17. Do you think that this item is unnecessary for safety before acupotomy?
(If there are more than two, please select multiple values.)
① Check the patient's identity and information (medication history, medical history, etc.).
② Identification of contraindications
③ Check vital signs (blood pressure, pulse, respiratory rate, body temperature)
④ Check the correct position according to the treatment site

[3] This section is supplemented with a more extensive explanation.

⑤ Indication of exact point site
⑥ Confirmation of anatomical safe treatment site
⑦ None

18. Do you think that this item is necessary for safety during acupotomy?
(If there are more than two, please select multiple values.)
① Check vital signs (blood pressure, pulse, respiratory rate, body temperature)
② Patient response observation (nerve stimulation symptoms, pain during procedure)
③ Assessment of the appropriateness of the procedure in terms of needling depth and blade direction
④ None

19. Do you think that this item is unnecessary for safety during acupotomy?
(If there are more than two, please select multiple values.)
① Check vital signs (blood pressure, pulse, respiratory rate, body temperature)
② Patient response observation (nerve stimulation symptoms, pain during procedure)
③ Assessment of the appropriateness of the procedure in terms of needling depth and blade direction
④ None

20. What items do you check for safety after acupotomy?
(Duplicate selection was allowed)
① Evaluation of nerve damage (motor, sensory deficit)
② Check blood vessel damage (bleeding, hematoma)
③ Evaluation of pain occurrence
④ Check vital signs (blood pressure, pulse, respiratory rate, body temperature)
⑤ Education on precautions, such as notification of possible pain and infection prevention methods

Perceptions about the workload and appropriate price for the acupotomy

21. What is the average time (in minutes) from preparation for acupotomy treatment (including disinfection, confirmation of imaging and safe treatment area) to post-procedure treatment and education on precautions, such as notification of possible pain and infection prevention methods?

[ ]

For example) if it is 1h and 20 min, you only need to enter "80."

22. Do you think how much acupotomy needs technical difficulty compared to acupuncture?

① Very high
② High
③ Neutral
④ Low
⑤ Very low

23. Do you think how much acupotomy needs physical burden compared to acupuncture?

① Very high
② High
③ Neutral
④ Low
⑤ Very low

24. In the current national health insurance system, the fee for acupotomy service is 1 part_4,429 won / 2 parts_6,216 won. Do you think that the current price of acupotomy is appropriate?

① Raise
② Suitable
③ Lower

25. How much should the acupotomy price (Korean won) be?

(If you choose 20,001 won or more, please describe the price you think is appropriate.)

① Current price-10,000 won
② 10,001-15,000 won
③ 15,001-20,000 won
④ 20,001 won or more: [ ]

Observed acupotomy-related adverse events and serious adverse events

This section pertains to the reporting of any adverse events or serious adverse events experienced after undergoing acupotomy. The survey is anonymous, and the collected data will be solely used for the purpose of identifying and documenting adverse events. We kindly request you to provide as much detail as possible while completing the questionnaire.[4]

* Definitions of adverse events and serious adverse events are as follows.
Adverse events: Any unexpected occurrences during or after acupotomy treatment
Serious adverse events: Adverse events that result in hospital admission or an extended hospital stay, permanent disability, death or disability, or life-threatening adverse events.

Filshie J, White A, Cummings M. Medical Acupuncture; A Western Scientific Approach. Seoul (Korea): Hanmi; 2019. 208p.

26. Please select the local adverse events that occurred after the acupotomy.
(Duplicate selection was allowed)
Patients with pain and bleeding that disappeared within 1-2 days were excluded from adverse events.[5]

① Pain
② Bleeding
③ Hematoma
④ Bruise
⑤ Blister
⑥ Neurological symptoms
⑦ Cerebrospinal fluid leakage
⑧ Organ damage
⑨ Pneumothorax
⑩ Local infections
⑪ Others (be specific): [ ]
⑫ None

27. Please select the systemic adverse events that occurred after the acupotomy.
(Duplicate selection was allowed)

① Autonomic nervous system dysfunction (headache, nausea, dizziness, and needle sickness)
② Fatigue
③ Psychiatric disorder (anxiety, impatient, etc.)
④ Systemic infection
⑤ None

28. What do you think are the main factors that contributed to the occurrence of the adverse events selected above?
(Duplicate selection was allowed)
However, if no adverse events occurred, select “None” and proceed.

① Insufficient identification of anatomically safe procedure zones
② Negligence or misconduct in the contraindication for acupotomy

---

4 This section is supplemented with a more extensive explanation.

5 The revisions incorporated are in response to feedback received.

③ Excessive number of up and down incision actions
④ Wrong blade direction
⑤ Incorrect needling depth
⑥ Needling speed
⑦ Improper insertion point selection
⑧ Negligence of disinfection and patient education (prevention of infection and pain control) after acupotomy
⑨ Blade width
⑩ Others (be specific): [ ]
⑪ None

29. Have you experienced any serious adverse events (hospital admission or extended hospital stay, permanent disability, death)?

① Yes
② No

30. What were the serious adverse events?
(If duplicate selection is necessary because it has occurred more than twice, please do so.)

① Pneumothorax
② Organ damage
③ Tissue damage at the treatment site
④ Central, peripheral nervous system damage
⑤ Cerebrospinal fluid leakage
⑥ Autonomic nervous system dysfunction (syncope, headache, nausea, dizziness, needle sickness, etc.)
⑦ Local infections
⑧ Systemic infections
⑨ Psychiatric disorder (anxiety, impatient, etc.)
⑩ Others (be specific): [ ]

31. Please select the two most significant factors that contributed to the occurrence of the serious adverse events chosen above.

① Insufficient identification of anatomically safe procedure zones
② Negligence or misconduct in the contraindication for acupotomy
③ Excessive number of up and down incision actions
④ Wrong blade direction
⑤ Incorrect needling depth
⑥ Needling speed
⑦ Improper insertion point selection
⑧ Negligence of disinfection and patient education (prevention of infection and pain control) after acupotomy
⑨ Blade width
⑩ Others (be specific): [ ]

32. Please select a result after serious adverse events occur.
(If duplicate selection is necessary because it has occurred more than twice, please do so.)

① Complete recovery
② Recovered, but sequelae occurred
③ Recovering
④ Not recovered
⑤ Death
⑥ Don't know

33. Please describe the number of days it took for the serious adverse events to disappear. [ ]

(If there are more than two occurrences, please indicate the maximum number of days).

Demographic information and other collected data

34. If there is anything you would like to say more about acupotomy that is not covered in this questionnaire, please write.
[ ]

35. Please write if you have any personal opinions or requirements when developing clinical guidelines for acupotomy treatment to be used in actual clinical practice.
[ ]

36. Please select your gender.

① Men

② Women

37. Please describe your age (international age). [ ]

38. Please describe the year of Korean medicine license acquisition. [ ]

39. Please select your work type.

① General or Korean medicine hospital
② General or Korean medicine clinic
③ Public health center
④ Others (be specific): [ ]

40. Please answer whether you are a board-certified specialist.

① General practitioner
② Current training (internship)
③ Current training (residency)
④ Western or Korean medicine multiple licenses
⑤ Specialist for internal medicine of Korean medicine
⑥ Specialist for acupuncture and moxibustion medicine
⑦ Specialist for rehabilitation medicine of Korean medicine
⑧ Specialist for obstetrics and gynecology in Korean medicine
⑨ Specialist for pediatrics of Korean medicine
⑩ Specialist for neuropsychiatry of Korean medicine
⑪ Specialist for ophthalmology, otorhinolaryngology, and dermatology of Korean medicine
⑫ Specialist for Sasang constitutional medicine

41. Please select the administrative district where you work.

[1] Seoul
[2] Gyeonggi
[3] Incheon
[4] Busan
[5] Daegu
[6] Daejeon
[7] Gwangju
[8] Ulsan
[9] Sejong
[10] Gangwon
[11] Chungcheongnam-do
[12] Chungcheongbuk-do
[13] Jeollanam-do
[14] Jeollabuk-do
[15] Gyeongsangnam-do

[16] Gyeongsangbuk-do
[17] Jeju
[18] Others (be specific): [   ]

42. Please select your working area.

① Urban

② Rural

Supplementary Table S1. Number of respondents per question.

Domains	Items (n)														
Part 1	Q1 (110)	Q2 (110)	Q3 (109)	Q4 (107)	Q5 (110)	Q6 (111)	Q7 (110)	Q8 (111)	Q9 (111)	Q10 (111)	Q11 (110)	Q12 (111)	Q13 (111)	Q14 (111)	Q15 (15)
Part 2	Q16 (110)	Q17 (107)	Q18 (110)	Q19 (110)	Q20 (110)										
Part 3	Q21 (110)	Q22 (110)	Q23 (110)	Q24 (111)	Q25 (109)										
Part 4	Q26 (110)	Q27 (111)	Q28 (111)	Q29 (110)	Q30 (2)	Q31 (2)	Q32 (2)	Q33 (2)							
Part 5	Q36 (109)	Q37 (109)	Q38 (109)	Q39 (109)	Q40 (109)	Q41 (109)	Q42 (109)								

n, number of respondents; Part 1, clinical usage status of acupotomy treatment; Part 2, perception of the clinical applicability of the "pre- and post-acupotomy procedure safety checklist" guidelines; Part 3, perceptions about the workload and appropriate price for the acupotomy; Part 4, observed acupotomy-related adverse events and serious adverse events; Part 5, demographic information, and other collected data; Q, Question number.

Supplementary Table S2. Pre- and post-acupotomy disinfection and treatment methods.

Question number	Disinfection and treatment methods		n (%)
Q10	Medical supplies for the prevention of infection related to acupotomy*		111 (100)
		Alcohol	99 (89.2)
		Povidone-iodine	82 (73.9)
		Surgical masks	76 (68.5)
		Surgical gloves	65 (58.6)
		Sterilization wraps	16 (14.4)
		Boric acid	3 (2.7)
		Surgical caps	1 (0.9)
		Antibiotics	1 (0.9)
		Surgical gowns	0 (0)
Q11	Treatment for pain relief*		110 (100)
		None	64 (58.2)
		Ice packs	28 (25.5)
		Surface anesthesia (anesthetic cream)	21 (19.1)
		Local anesthesia with conventional anesthetics (lidocaine, etc.)	6 (5.5)
		Oral herbal analgesics	6 (5.5)
		Non-steroidal anti-inflammatory drugs	4 (3.6)
		Local anesthesia with herbal anesthetics (Sumsu pharmacopuncture, etc.)	3 (2.7)
Q12	Post-procedure care*		111 (100)
		Disinfection and sterilization	98 (88.3)
		Band-aid application	86 (77.5)
		Compression for hemostasis	69 (62.2)
		Dressing with sterile gauze	24 (21.6)
		Cupping	3 (2.7)
		Ice pack application	1 (0.9)

* Duplicate selection was allowed.

Supplementary Table S3. Comparison of practice features according to career years of acupotomy practice.

Question number	Features	Beginners[†]	Experts[‡]	t	p-value
Q7	Number of up and down incision actions per point[§]	3.79 ± 2.48	2.92 ± 1.53	2.172	0.032[*]
Q5	Depth of insertion[‖]	58 (100)	51 (100)		
	Epidermis	5 (8.6)	2 (3.9)		
	Muscle	38 (65.5)	22 (43.1)		0.014[*]
	Periosteum	15 (25.9)	27 (52.9)		
Q9	The average number of acupotomy sessions in one week[‖]	58 (100)	52 (100)		
	1-5	23 (39.7)	9 (17.3)		
	6-10	11 (19)	7 (13.5)		
	11-20	12 (20.7)	8 (15.4)		
	21-30	7 (12.1)	9 (17.3)		0.009[*]
	31-40	2 (3.4)	4 (7.7)		
	41-50	2 (3.4)	7 (13.5)		
	51≤	1 (1.7)	8 (15.4)		

Q, Question number.
Statistics are expressed as mean ± standard deviation and number (percent).
[*] Statistically significant ($p<0.05$).
[†] Less than 3 career years of acupotomy practice.
[‡] More than 4 career years of acupotomy practice.
[§] Independent t-test was used.
[‖] Fisher's exact test was used for statistical analysis.

Supplementary Table S4. Perception on the appropriateness of items in the "pre- and post-acupotomy procedure safety checklist".

Question number	Pre- and post-acupotomy procedure safety checklist	n (%)
Q16	Do you think that this item is necessary for safety before acupotomy?*	110 (100)
	Anatomical safe treatment site	91 (82.7)
	Patient identity	73 (66.4)
	Identification of contraindications	73 (66.4)
	Indication of exact point site	55 (50)
	Position	52 (47.3)
	Vital signs	47 (42.7)
	None	0 (0)
Q17	Do you think that this item is unnecessary for safety before acupotomy?*	107 (100)
	None	77 (71.2)
	Vital signs	13 (12.2)
	Patient identity	10 (9.4)
	Indication of exact point site	8 (7.5)
	Identification of contraindications	7 (6.5)
	Position	7 (6.5)
	Anatomical safe treatment site	2 (1.9)
Q18	Do you think that this item is necessary for safety during acupotomy?*	110 (100)
	Assessment of the appropriateness of the procedure in terms of needling depth and blade direction	92 (83.6)
	Patient response observation	90 (81.8)
	Vital signs observation	50 (45.5)
	None	1 (0.9)
Q19	Do you think that this item is unnecessary for safety during acupotomy?*	110 (100)
	None	93 (84.6)
	Vital signs observation	12 (10.9)
	Patient response observation	5 (4.5)
	Assessment of the appropriateness of the procedure in terms of needling depth and blade direction	5 (4.5)
Q20	What items do you check for safety after acupotomy?*	110 (100)
	Blood vessel damage (bleeding, hematoma)	102 (92.7)
	Patient education (pain control, preventing infection)	87 (79.1)
	Pain	69 (62.7)
	Nerve damage (motor, sensory deficit)	53 (48.2)
	Vital signs	21 (19.1)

* Duplicate selection was allowed.

Supplementary Table S5. Perceptions about the workload and appropriate price for the acupotomy.

Question number	Workload and appropriate price		n (%)	Mean ± SD
Q21	Procedure duration (minutes)			23.64 ± 18.68
Q22	Technical difficulty		110 (100)	
		Very high	40 (36.4)	
		High	63 (57.3)	
		Neutral	7 (6.3)	
		Low	0 (0)	
		Very low	0 (0)	
Q23	Physical burden		110 (100)	
		Very high	23 (20.9)	
		High	57 (51.8)	
		Neutral	27 (24.6)	
		Low	3 (2.7)	
		Very low	0 (0)	
Q24	Perception of the current price levels		111 (100)	
		Raise	109 (98.2)	
		Suitable	3 (1.8)	
Q25	Appropriate price (KRW)		109 (100)	
		≤10,000	5 (4.6)	
		10,001-15,000	14 (12.8)	
		15,001-20,000	28 (25.7)	
		20,001-30,000	19 (17.4)	
		30,001-40,000	8 (7.3)	
		40,001-50,000	28 (25.7)	
		50,001-60,000	3 (2.8)	
		70,000	1 (0.9)	
		100,000	2 (1.8)	
		150,000	1 (0.9)	

KRW, Korean Won; SD, Standard deviation.

Supplementary Table S6. Comparison of the workload of Korean medicine doctors and appropriate prices according to career years in acupotomy practice.

Question number	Workload and appropriate price	Beginners†	Experts‡	p-value
Q22	Technical difficulty	57 (100)	52 (100)	
	Very high	16 (28.1)	24 (46.2)	0.144
	High	37 (64.9)	25 (48.1)	
	Neutral	4 (7)	3 (5.8)	
Q23	Physical burden	57 (100)	52 (100)	
	Very high	6 (10.5)	17 (32.7)	0.005*
	High	30 (52.6)	26 (50)	
	Neutral	20 (35.1)	7 (13.5)	
	Low	1 (1.8)	2 (3.8)	
Q25	Appropriate price (KRW)	57 (100)	51 (100)	
	≤10,000	5 (8.8)	0 (0)	0.018*
	10,001-15,000	11 (19.3)	3 (5.9)	
	15,001-20,000	13 (22.8)	15 (29.4)	
	20,001≤	28 (49.1)	33 (64.7)	

KRW, Korean Won; Q, Question number.
Fisher's exact test was used.
* Statistically significant ($p<0.05$).
† Less than 3 career years of acupotomy practice.
‡ More than 4 career years of acupotomy practice.

Supplementary Table S7. Use of medical imaging devices.

Question number	Use of medical imaging devices		n (%)
Q13	Do imaging devices exist in your hospital (or clinic)?		111 (100)
		Yes	28 (25.2)
		No	83 (74.8)
Q14	Are you using imaging devices during acupotomy?		111 (100)
		Yes	15 (13.5)
		No	96 (86.5)
Q15	Type of imaging devices		15 (100)
		C-arm	12 (80)
		X-ray	2 (13.3)
		SONO	1 (6.7)
		CT	0 (0)
		MRI	0 (0)

CT, Computed tomography; MRI, Magnetic resonance angiography; SONO, Sonography.

Supplementary Table S8. Comparison of the number of adverse events according to the imaging device used.

	Using imaging device (n = 15)	Not using (n = 96)	t	p-value
Number of local AEs	2.32 ± 1.18	3.13 ± 1.13	-2.511	0.014*
Number of systemic AEs	0.86 ± 0.71	1.13 ± 0.74	-1.363	0.176
Total number of AEs	3.20 ± 1.48	4.27 ± 1.34	-2.611	0.010*

AEs, Adverse events; n, number of respondents.
An Independent t-test was used.
Statistics are expressed as mean ± standard deviation.
* Statistically significant ($p<0.05$).

Supplementary Table S9. Clinicians' demands for the safety of acupotomy.

**Demands**
The need for system improvement and sufficient insurance coverage
"I think the price should be further increased as the inclusion of the second body part is standard."<br>*"I wish the actual transaction price of acupotomy medical supplies for disinfection could be accurately reflected."*<br>*"Health insurance fee and mandatory registration of new medical technology."*<br>*"Mandatory registration of new medical technology is necessary."*
Professional training time required
"It would be beneficial to consider the completion time of training for acupotomy."<br>*"I hope doctors have undergone comprehensive anatomical knowledge and technical training for acupotomy."*<br>*"There is a need for education regarding proper surgical posture, direction, and depth."*<br>*"Acupotomy should be performed after receiving training at the specialist or master level."*<br>*"Anatomical education should be more comprehensive and detailed."*
Need to enhance blade sharpening and develop thinner needle tips
"Enhancing the dissemination of thin needle tips."<br>*"Various acupotomy tools should be developed to meet diverse needs."*<br>*"Improvement in blade sharpening techniques for needle production is necessary."*
The necessity of using medical imaging devices
"Using medical imaging devices during the procedure is necessary."<br>*"It would be advantageous to have access to medical imaging devices."*
Development of several practically usable versions of the acupotomy clinical guidelines
"It is necessary to create a streamlined version highlighting only the crucial elements, as well as a comprehensive version. Both versions cater to different needs."<br>*"We require guidelines that accurately represent the practical aspects of the field."*
The necessity of describing differences in the degree of adverse events by patient
"Individuals who are slim and delicate may experience difficulties or increased body soreness during acupotomy treatment."<br>*"Assessing the severity of adverse events based on the patient's body type."*
The necessity of describing the safe depth of insertion for each treatment site
"To provide comprehensive guidelines encompassing safe insertion depth, appropriate stimulation techniques, and other important instructions."

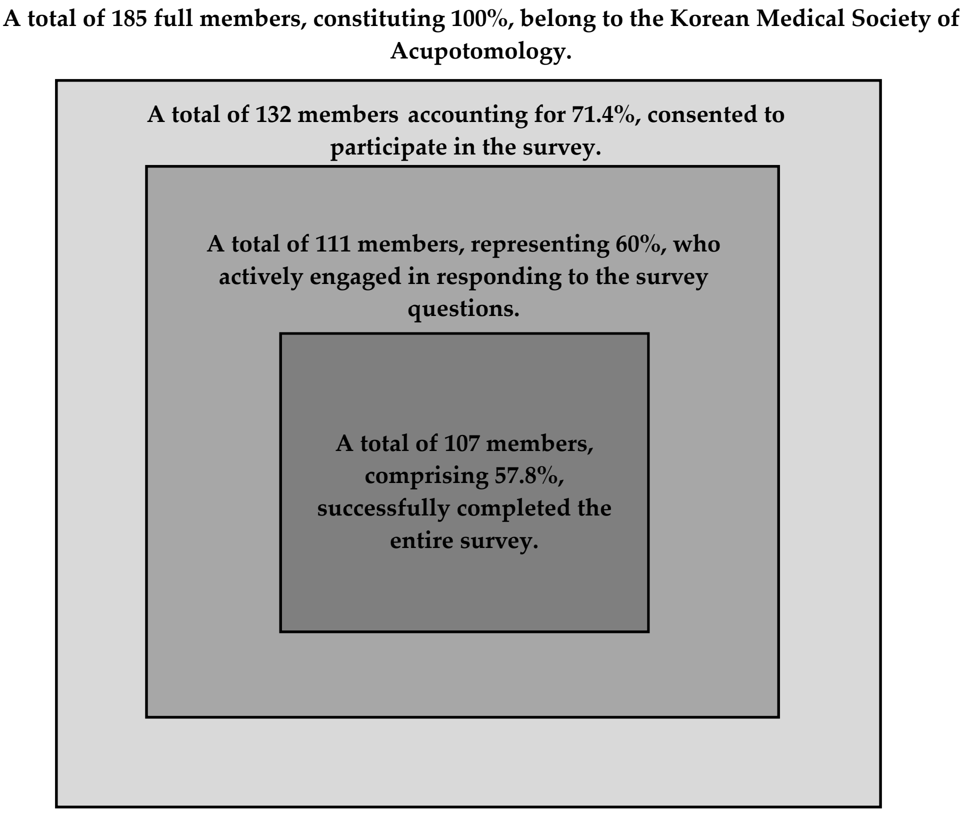

Supplementary Figure S1. Count of survey respondents and the corresponding response rate.

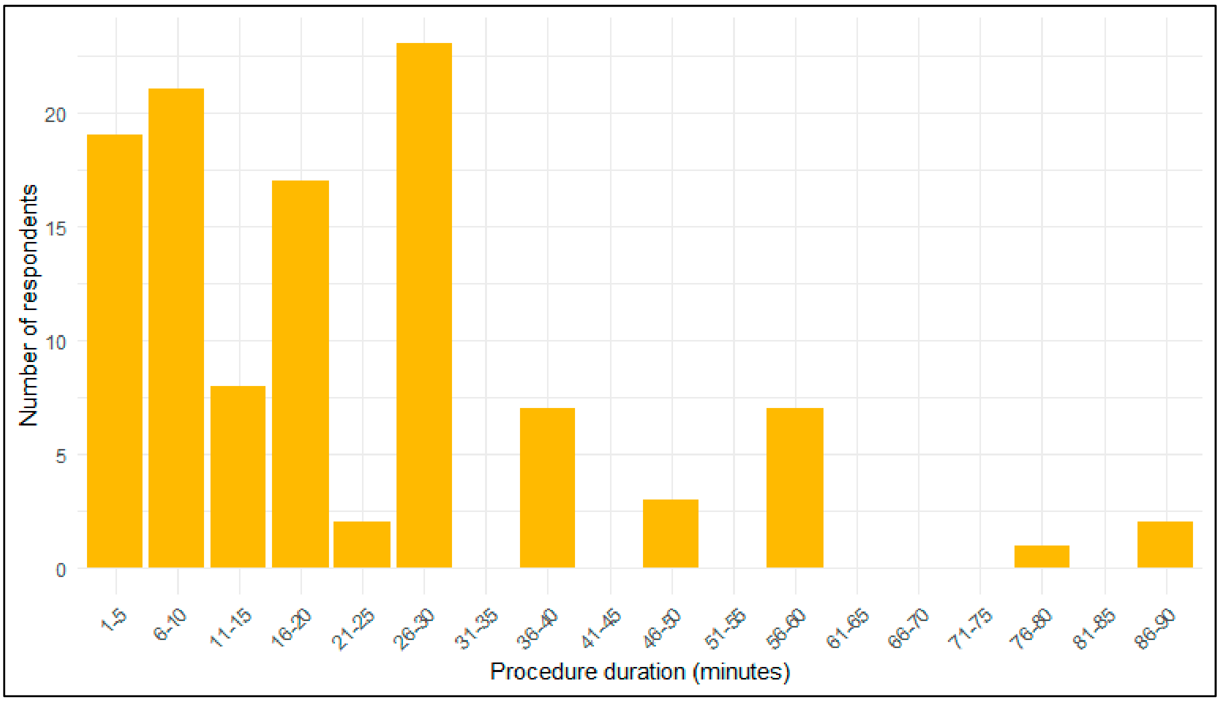

Supplementary Figure S2. A bar plot depicting the number of respondents based on procedure duration minutes.

(Q21) Procedure duration (minutes)
